# Supplementary material for: Effects of Adipose-Derived Stem Cells and Their Conditioned Medium in a Human Ex Vivo Wound Model
Source: Cells. 2022 Apr 2;11(7):1198. doi: 10.3390/cells11071198 (PMC8998073; doi:10.3390/cells11071198)
Supplement: Supplementary file 1 [file cells-11-01198-s001.zip › cells-1640994-supplementary.pdf]

Supplemental Material

Effects of Adipose-derived Stem Cells and Their Conditioned Medium in a Human ex vivo Wound Model

Xiao Guo [1], Christoph Schaudinn [2], Ulrike Blume-Peytavi[1], Annika Vogt[1] and Fiorenza Rancan [1,*]

[1]Clinical Research Center for Hair and Skin Science, Department of Dermatology and Allergy, Charité-Universitaetsmedizin Berlin, corporate member of Freie Universität Berlin, Humboldt-Universität zu Berlin, and Berlin Institute of Health, Germany; xiao.guo@charite.de
[2]Advanced Light and Electron Microscopy, ZBS4, Robert Koch Institute, Berlin 13353, Germany; schaudinnc@rki.de
*Correspondence: fiorenza.rancan@charite.de; Tel.: + 49-30-450518347

β-catenin expression

The expression of β-catenin was measured in wound tissue extracts by means of ELISA (Invitrogen Corporation, Carlsbad, CA, USA). Tissue from four donors was frozen in liquid nitrogen, 20 x 50 µm-thick horizontal sections were prepared and extracted. The amounts of β-catenin were normalized to the amount of total protein (TP) in the respective sample. A time dependent decrease of β-catenin was measured. With exception for day 3, no significant differences were measured between the groups.

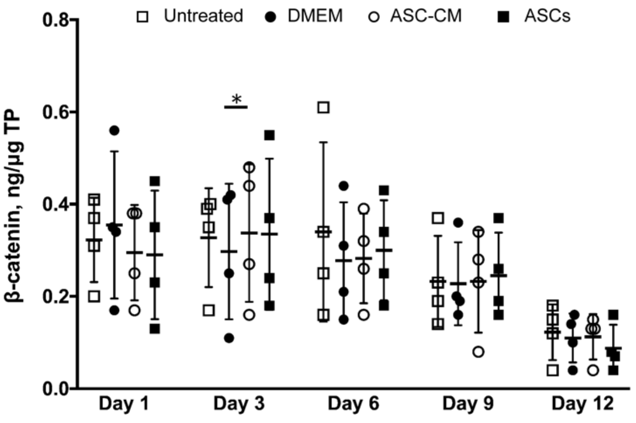

Figure S1. Quantification of β-catenin in whole skin extracts. β-catenin expression following topical treatments of DMEM (10% FCS), ASC-CM, and ASCs. Values were normalized to total protein. Means ± SD are shown. Statistical analysis used Student's t-test. * $p < 0.05$.

IL-6 and IL-8 Expression

In preliminary experiments, IL-8 and IL-6 expression were measured in samples treated with MDEM (10% FCS) and ASCs. The culture medium was refreshed every three days and samples were collected at day 3, 6, 9, and 12 (Fig. S2). Skin samples were frozen and horizontal sections were cut and divided in epidermis (5 x 20 µm-thick sections) and dermis (18 x 50 µm-thick sections). Cytokines were measured in culture media and extracts by means of ELISA (Invitrogen Corporation, Carlsbad, CA, USA) and normalized to total protein (Fig. S3). IL-6 and IL-8 increased over time. Significant increment of IL-6 in the ASCs group with respect to the DMEM group was measured in the dermis at day 6. Significant increments of IL-8 in the ASCs group with respect to the DMEM group were measured in the epidermis from day 3 to day 12.

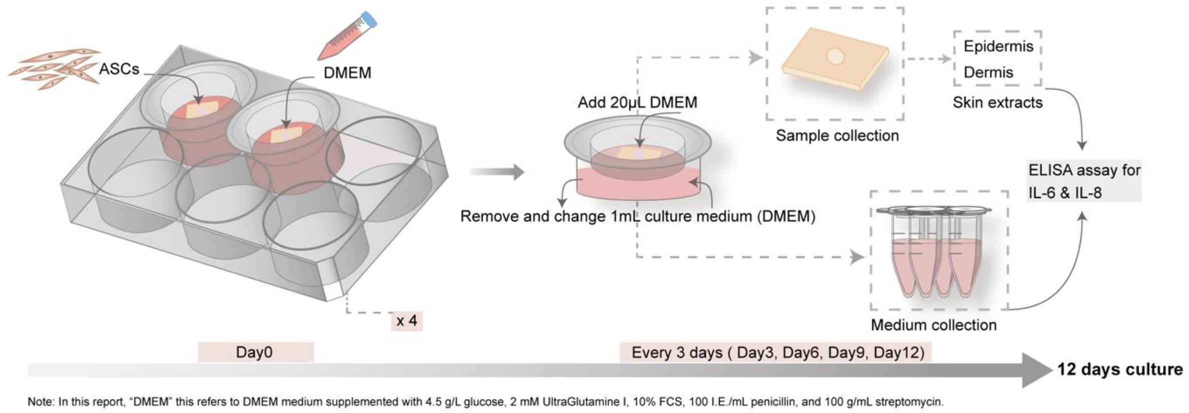

Figure S2. Schematic of the experimental protocol in preliminary experiments with the *ex vivo* wound model.

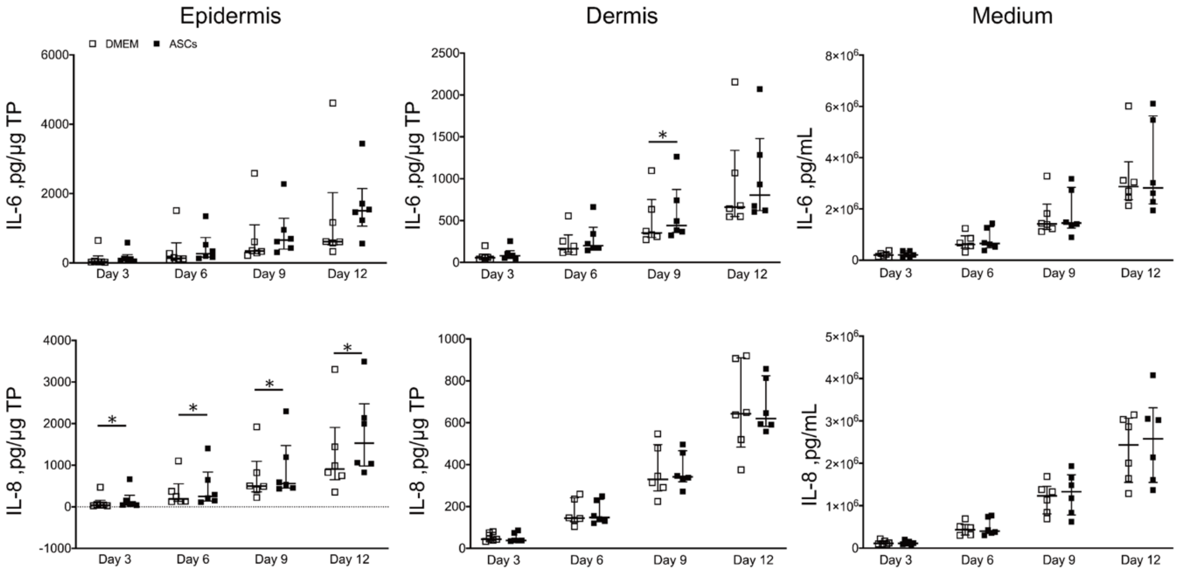

Figure S3. IL-6 and IL-8 expression following topical treatments of DMEM and ASCs. Values from epidermis and dermis were normalized to total protein and presented as cumulative amounts over the incubation time. Median with IQR are also shown. Statistical analysis was done with the Wilcoxon signed-rank test. * $p < 0.05$.
